# Rating Health Web sites using the principles of Citation Analysis: A Bibliometric Approach

**DOI:** 10.2196/jmir.1.1.e4

**Published:** 1999-08-05

**Authors:** Lei Cui

**Keywords:** Web sites, citation analysis, health science, evaluation, bibliometric

## Abstract

The rapid growth in the number of health care related web sites necessitates that medical librarians be able to evaluate the quality of the web sites. By analysing the linked sources medical libraries web pages of nineteen of the top U.S. medical schools, this study used the citation analysis method. What was found with this bibliometric approach was a set of 78 most highly cited WWW sites out of thousands of cited links. The identification of the current, core section of health sciences related web sites with a bibliometric method gives librarians and information scientists another approach for evaluating web sites.

## Introduction

The rapid growth and constant change in the number of health care related web sites make the evaluation of the quality of the web sites a difficult but beneficial task. The Internet is "a medium in which anyone with a computer can serve simultaneously as author, editor, and publisher and can fill any or all of these roles anonymously if he or she so choose. In such an environment, novices and savvy Internet users alike can have trouble distinguishing the wheat from the chaff, the useful from the harmful" [1]. In a systematic search by means of two search engines (Yahoo and Excite) for parent-oriented web pages relating to home management of feverish children, [2] the investigators of this study, compared the web site information with the guidelines to parents for managing fever at home supplied by a printed book. The investigators found among 41 web pages retrieved and reviewed: 28 web pages gave a specific temperature above which a child is feverish, 26 pages indicated the optimal site for taking temperature, 38 pages recommended non-drug measures, and 36 pages gave some indication of when a doctor should be called. Only four web pages adhered closely to the main recommendations in the guidelines. The investigators concluded from these observations only a few web sites provided complete and accurate information for this common and widely discussed condition. According to McClung, [3] 48 out of 60 major medical institution web sites checked had inaccurate information about the treatment of childhood diarrhea. While it is virtually impossible (and probably undesirable) to control the content of web pages, it is certainly useful to have some measure of the quality of the information provided.

One possibility is to establish an official rating system based on standard criteria. In the survey mentioned above, [2] the author also suggested an urgent need to check public oriented health care information on the Internet for accuracy, completeness, and consistency. Many attempts have been made, and core standards that can help to achieve these goals have been developed. The most widely accepted suggestion is adapting the five traditional print evaluation criteria: accuracy, authority, objectivity, currency and coverage, to web resources [4,5,6].

However, "many Internet users object strongly to any 'official' attempts to regulate information", though few want to see inaccurate information appearing! In addition, "the Web's interactive format means criteria used for paper-based journals may not be valid for web-based information." [7]. Jadad points out that the "Net's very nature makes this difficult, if not impossible". After an investigation to identify instruments used to rate web sites providing health information on the Internet, Jadad concluded, "many incompletely developed instruments to evaluate health information exist on the Internet. It is unclear, however, whether they should exist in the first place, whether they measure what they claim to measure, or whether they lead to more good than harm" [8]. At this point it is very difficult to reach or develop a standard that every user of the Internet could observe.

It has been suggested that Web sites can be evaluated in a similar way to traditional print media. When we evaluate a textbook or a journal, we not only assess the authors, content, and structure, but also more objectively, measure the impact of the publication on its readers. Citation analysis, the practice of counting citations to determine the scholarly impact of a work, is a method long used by librarians as an important tool of collection development. With bibliometrics the impact of a journal is evaluated by the frequency that it was cited during a certain period.

One major instrument to evaluate scientific journals is Journal Citation Reports (JCR) [9]. JCR is published by the Institute for Scientific Information and includes several citation-based measures of journal impact for the journals that they review. Librarians and researchers can utilize JCR to see how many times and how quickly articles published in certain journals are cited. There is also a measure of effectiveness, the impact factor, which normalizes the citations received by the selected journals and looks only at the previous two years of publication.

Though there is no similar tool available to evaluate the impact of a WWW page, it is comparatively easy to determine which pages are cited ("linked to") by the compilers of other pages. We also found a study conducted on the WWW pages of selected fine art libraries [10]. By analyzing the linked sources on art library Web pages, Neth's study found a set of twenty commonly cited WWW sites out of thousands of cited links. As we investigate health science related web sites, we also find some well-established sites already use this method successfully. For example the compilers for the Hardin-Meta of the University of Iowa look at many sites in each field and chose the lists that are most frequently cited by people in the field. This analysis provides a rudimentary form of peer evaluation. They call it a "list of lists" [11]. Another example, in a paper on the quality management of medical information on the Internet by Eysenbach, the author presented some indirect quality indicators, among them is "Web citation". A "webcite index," analogous to the Science Citation Index, could be compiled from the absolute number of hyperlinks to a certain website or new hyperlinks established over a period of time [17]. The author has developed a website network (http://webcite.net) contributing and practicing this methodology.

In this paper, we analysis the pages linked to in the "other links" sections of the web pages of a selection of the top 25 US medical schools. On the assumption that a Web Master will only cite or link to pages he/she thinks are authoritative. We examine the links made from these pages and obtain a listing of the most cited pages. This affords a new approach to evaluate web sites by using the principles of citation analysis.

## Methods

(1). Sample selection:

The selection of the "key sites" used to count the most frequently cited web sites is very important. For our approach, we used the listing of "the top 25 medical schools in the United States" as published by U.S. News and World Report [12]. Next we identified their primary health information WWW site. Normally this was the home page for the medical school library. Among these 25 medical schools, the web pages for seven of the medical schools were eliminated due to technical limitation of the URL checking software and the variations of the Web sites.

We finally examined the web pages of nineteen of the top twenty-five US medical schools. The top 25 are listed in Table 1 with those eliminated from this study indicated by an asterisk (*).

**Table 1:** The top 25 Medical Schools and Their Web Pages Used in Survey

Medical Schools	Page Used in Survey
Harvard University (MA) Johns Hopkins University (MD) Washington University (MO) Duke University (NC) University of Pennsylvania Yale University (CT)* Columbia University College University of California-SF * Cornell University (NY) Stanford University (CA) University of Michigan-AA University of California-LA Baylor College of Medicine (TX) University of Washington * Case Western Reserve University Vanderbilt University (TN)* University of Texas SW Medical Center-Dallas University of Chicago University of California--SD University of Pittsburgh* Emory University (GA)* New York University Mayo Medical School (MN) Yeshiva Univ.-Albert Einstein Coll. of Medicine	http://www.countway.harvard.edu/countway/webref/catalog.html http://www.welch.jhu.edu/internet/ http://medschool.wustl.edu/~ref/otherwww.htm http://www.mc.duke.edu/mclibrary/practice/ http://www.library.upenn.edu http://www.med.yale.edu/library/sir/ http://cpmcnet.columbia.edu/library/subject/ http://www.library.ucsf.edu/kr/bin/topics.pl http://www.med.cornell.edu/CUMC/links.html Http://www-med.stanford.edu/lane/bioresources.html http://www.lib.umich.edu/libhome/Taubman.lib/webresources.html http://www.library.ucla.edu/libraries/biomed/cdd/list.htm http://www.library.tmc.edu/selected.html http://www.hslib.washington.edu/ http://www.cwru.edu/chsl/catalogs.htm http://www.mc.vanderbilt.edu/library/resources.html http://www.swmed.edu/home_pages/library/rcis/intro.htm http://www.lib.uchicago.edu/LibInfo/Internet/ http://scilib.ucsd.edu/bml http://www.hsls.pitt.edu/intres/index.html http://www.cc.emory.edu/WHSCL/medweb.html http://library.med.nyu.edu/library/internet/biomedical/biosubjects.html http://www.mayo.edu/outlinks.html http://bagel.aecom.yu.edu/

(2). Ranking the web sites by the cited frequency

The next step was to examine the links made from these pages. This was achieved by using a software program "Checkweb" [13], which checks the links of the selected web page and reports which ones have moved, or cannot be located or connected to. The second step is to clean up this list of and eliminate the orphans (Status 404 - no longer existing and Status 301 and 302 - moved), and the "noise items". Noise items are "noise" from the host web page such as "go home" or links to other sections on the same site. This ensures that the final list is only to active links to external URLs.

The final step was to count the frequency of these URLs by their different levels. For example, we have the URLs such as: http://www.lib.uiowa.edu/hardin/md/speech.html. This URL can be broken down into its component parts as shown in Table 1. We separated these URLs into their different component levels and counted their frequency. In this example, the first level domain name is the portion before the first slash, "http://www.lib.uiowa.edu".

The Top Level Domains (TLDs) include the designators such as .edu, .com, .ca, and .nl. Sorting the TLDs resulted in Table 2.

**Table 2:** The distribution of the Top Level Domains (TLDs)

**No.**	**TLD**	**Meaning of TLDs**	**Freq.**	**Percen**	**Cum.P**
1	edu	U.S. Four year colleges and universities	1124	30.47	30.47
2	com	U.S. Commercial entities	839	22.74	53.21
3	gov	United States Federal Government entities	683	18.51	71.73
4	org	Miscellaneous organisations	623	16.89	88.61
5	net	Organisations directly involved in Internet operations	93	2.52	91.14
6	uk	United Kingdom	83	2.25	93.39
7	ca	Canada	47	1.27	94.66
8	ch	Switzerland	33	0.89	95.55
9	de	Germany	24	0.65	96.20
10	us	United States	18	0.49	96.69
11	au	Australia	16	0.43	97.13
12	se	Sweden	10	0.27	97.40
13	jp	Japan	9	0.24	97.64
14	fr	France	8	0.22	97.86
15	int	International	8	0.22	98.08
16	it	Italy	6	0.16	98.24
17	nl	Netherlands	5	0.14	98.37
18-52		TLDs appear less than 5 times	60	1.63	100.00
Total	52		3689	100.00	

## Results and Analysis

The three levels of URLs were counted and the results are shown in Table 3,Table 4 and Table 5.

(1). The Top Level Domains distribution.

The frequency of links is very concentrated in several TLDs, notably .edu, .com, and .gov and. org. These accounted for 88.61% of the Links.

As shown in Table 3 the most highly cited TLDs (greater than 600 times) are .edu, .com, .gov, and .org. These TLD's are all registered in the United States. Other less cited US TLDs are .net, .us and .mil. The United States related web pages account for almost 90% of the URLs cited. This was not unexpected because the source samples are U.S. medical schools and the Internet is highly developed in this country. Among the US TLDs, those from four years colleges and universities, those entitled to use the .edu suffix, are cited most frequently and therefore are considered the most important. The .edu suffix accounts for almost one third of all links.

Other countries whose TLDs are frequently cited are United Kingdom (uk), Switzerland (ch), Canada (ca), Germany (de), Australia (au), Sweden (se) and Netherlands (nl). This distribution is very similar to the results of 30 nations ranked by the citations per paper from 1992 to 1996 by Institute of Scientific Information (ISI) published in the Science Watch [14]. In this study the top ten nations were Switzerland, United States, Netherlands, Sweden, Denmark, United Kingdom, Belgium, Finland, Canada, and Germany. It seems that in some degree our results may also represent the developmental level of medical information publishing and research in the world. However, the focus of this paper is not placed on the comparison of these two lists.

**Table 3:** The URLs and the frequency distribution of the "First Level Domains"

Rank	URLs	Freq	Percent	Cum Percent
1	http://www.yahoo.com	91	2.47	2.47
2	http://www.gen.emory.edu	86	2.33	4.80
3	http://www.cdc.gov	65	1.76	6.56
4	http://www.ama-assn.org	52	1.41	7.97
5	http://www.medmatrix.org	36	0.98	8.95
6	http://text.nlm.nih.gov	33	0.89	9.84
7	http://www.nih.gov	32	0.87	10.71
8	http://www.lib.uiowa.edu	28	0.76	11.47
9	http://galaxy.einet.net	27	0.73	12.20
10	http://www.aamc.org	26	0.70	12.90
11	http://www-sci.lib.uci.edu	26	0.70	13.61
12	http://www.ncbi.nlm.nih.gov	25	0.68	14.29
13	http://roger.ucsd.edu	21	0.57	14.85
14	http://www.nlm.nih.gov	21	0.57	15.42
15	http://www.slackinc.com	20	0.54	15.97
16	http://www3.ncbi.nlm.nih.gov	19	0.52	16.48
17	http://www.faseb.org	18	0.49	16.97
18	http://www.nsf.gov	17	0.46	17.43
19	http://golgi.harvard.edu	17	0.46	17.89
20	http://www.census.gov	16	0.43	18.32
21	http://pharminfo.com	15	0.41	18.73
22	http://indy.radiology.uiowa.edu	15	0.41	19.14
23	http://www.einet.net	14	0.38	19.52
24	http://www.bis.med.jhmi.edu	14	0.38	19.90
25	http://www.who.ch	14	0.38	20.28
26	http://www.pitt.edu	13	0.35	20.63
27	http://www.epa.gov	13	0.35	20.98
28	http://www.os.dhhs.gov	13	0.35	21.33
29	http://expasy.hcuge.ch	12	0.33	21.66
30	http://www.lycos.com	12	0.33	21.98
31	http://webcrawler.com	12	0.33	22.31
32	http://www.ornl.gov	12	0.33	22.63
33	http://www.altavista.digital.com	12	0.33	22.96
34	http://asmusa.edoc.com	11	0.30	23.26
35	http://www.ohsu.edu	11	0.30	23.56
36	http://neuro-www.mgh.harvard.edu	11	0.30	23.85
37	http://www.vh.org	11	0.30	24.15
38	http://vm.cfsan.fda.gov	11	0.30	24.45
39	http://gdbwww.gdb.org	11	0.30	24.75
40	http://vh.radiology.uiowa.edu	11	0.30	25.05
41	http://www.pslgroup.com	10	0.27	25.32
42	http://www.fda.gov	10	0.27	25.59
43	http://www.cc.emory.edu	10	0.27	25.86
44	http://chablis.cos.com	10	0.27	26.13
45	http://www.hcfa.gov	10	0.27	26.40
46	http://www.access.gpo.gov	10	0.27	26.67
47	http://www.med.upenn.edu	10	0.27	26.94
48	http://www.apa.org	10	0.27	27.22
49	http://www.merck.com	10	0.27	27.49
50	http://www.excite.com	10	0.27	27.76
51	http://www.clearinghouse.net	9	0.24	28.00
52	http://hiru.mcmaster.ca	9	0.24	28.25
53	http://www.dejanews.com	9	0.24	28.49
54	http://molbio.info.nih.gov	9	0.24	28.73
55	http://www.upenn.edu	9	0.24	28.98
56	http://www.hotbot.com	9	0.24	29.22
57	http://www.ebi.ac.uk	8	0.22	29.44
58	http://neurosurgery.mgh.harvard.edu	8	0.22	29.66
59	http://infonet.welch.jhu.edu	8	0.22	29.87
60	http://www.metacrawler.com	8	0.22	30.09
61	http://www2.infoseek.com	8	0.22	30.31
62	http://ificinfo.health.org	8	0.22	30.52
63	http://web.fie.com	8	0.22	30.74
64	http://www.who.int	8	0.22	30.96
65	http://www.lib.umich.edu	8	0.22	31.17
66	http://www.mckinley.com	8	0.22	31.39
67	http://atsdr1.atsdr.cdc.gov	8	0.22	31.61
68	http://www.medscape.com	7	0.19	31.80
69	http://info.cas.org	7	0.19	31.99
70	http://cancer.med.upenn.edu	7	0.19	32.18
71	Gopher://gopher.nih.gov	7	0.19	32.37
72	http://cancernet.nci.nih.gov	7	0.19	32.56
73	http://www.mgh.harvard.edu	7	0.19	32.75
74	http://lcweb.loc.gov	7	0.19	32.94
75	http://www2.nas.edu	7	0.19	33.13
76	http://www.ed.gov	7	0.19	33.32
77	http://fdncenter.org	7	0.19	33.51
78	http://www.osha.gov	7	0.19	33.69
79-1731	Less than 7 times (1653 URLs)	2464	66.31	100.00
Total	1731	3689	100.00	

(2). Distribution of the First Level Domains.

One of the goals of this study was to identify the web sites cited most frequently by US academic health sciences libraries. Table 3 shows that a total of 1731 web sites were cited by (linked to) these 19 institutional home pages.

According to the Bradford 's Law of Scatter: [15] "if scientific journals are arranged in order of decreasing productivity of articles on a given subject, they may be divided into a nucleus of periodicals more particularly devoted to the subject and several groups or zones containing the same number of articles as the nucleus, when the numbers of periodicals in the nucleus and succeeding zones will be as 1:n:n2 ". In our study, we list the web sites in order of decreasing frequency of citation, and as Bradford has done in his original paper we divide the total cited times of the web sites into 3 equal sections. The first section is the top 78 web sites (as shown in details in Table 3) 33.69% of total cited times, the second section is from rank No. 79 to No.530, nearly another 33% of total cited times, the last section is No. 531 to No. 1731. So the numbers of these web sites with almost equal cited frequency is 78:452:1201, close to 1:4:42. Thus by application of this law in the web sites citation analysis, we can take the first section (78 web sites) as a core section of these 1731 web sites.

(3). Distribution of the Whole Domain Name Web sites:

Most of the web sites listed in the whole domain name table (Table 4) are already listed in the earlier tables. This is because most "other links" are directed to the first level domains of URLs. Only URLs with asterisks (*) in this table have more details.

In fact, most of the whole URLs list were already been identified in the "First Level Domain" (Table 3), as most of the whole URLs are also represented in the "first level domain". A few links found are to pages deeper into the site and give us information as to why a site was selected for a link. For example, many, though not all of, visitors to CDC want to look up the Morbidity and Mortality Weekly Report (MMWR) and many visitors to NIH want information on grant and fellowship programs, both pages thus often get direct links in addition to a more general link to the CDC or NIH sites.

**Table 4:** The frequent distribution of the whole URLs

Rank	URLs	Freq	Percent	Cum. Percentage
1	http://www.medmatrix.org/index.asp/	33	1.01	1.01
2	http://www.nih.gov/	14	0.43	1.44
3	http://www.cdc.gov/	12	0.37	1.81
4	http://chablis.cos.com/	10	0.31	2.11
5	http://www.hcfa.gov/	9	0.28	2.39
6	http://www.nsf.gov/	9	0.28	2.66
7	http://www.nlm.nih.gov/	8	0.24	2.91
8	http://www.nih.gov/grants/	8	0.24	3.15
9	http://www3.ncbi.nlm.nih.gov/omim/	8	0.24	3.40
10	http://www.os.dhhs.gov/	8	0.24	3.64
11	http://www.cdc.gov/epo/mmwr/mmwr.html/	8	0.24	3.89
12	http://www.osha.gov/	7	0.21	4.10
13	http://www.ohsu.edu/cliniweb/wwwvl/	7	0.21	4.32
14	http://gdbwww.gdb.org/	7	0.21	4.53
15	http://atsdr1.atsdr.cdc.gov/	7	0.21	4.75
16	http://pharminfo.com/	6	0.18	4.93
17	http://www.ncbi.nlm.nih.gov/	6	0.18	5.11
18	http://savvy.cs.colostate.edu/	6	0.18	5.30
19	http://cancer.med.upenn.edu/	6	0.18	5.48
20	http://neuro-www.mgh.harvard.edu/hospitalweb.nclk/	6	0.18	5.66
21	http://www.epa.gov/	6	0.18	5.85
22	http://www.yahoo.com/health/medicine/	6	0.18	6.03
23	http://www.cdc.gov/nchswww/nchshome.htm/	6	0.18	6.22
24-39	Five(15)	75	2.30	8.51
40-56	Four (16)	64	1.96	10.47
57-125	Three (68)	204	6.25	16.72
126-450	Two (324)	648	19.84	36.56
451-2523	One (2072)	2072	63.44	100.00
	Total	3266	100.00	

**Table 5:** The categories of the most cited health-related core web sites

**1.Specialty Databases or Servers:**	
**1.1 University Original:**	
Science and Mathematics Resources, University of California, Irvine	http://www-sci.lib.uci.edu
Biological Links, Department of Molecular and Cellular Biology, Harvard University	http://golgi.harvard.edu
The Johns Hopkins University BioInformatics Web Server	http://www.bis.med.jhmi.edu
Gateway to Neurology at MGH	http://neuro-www.mgh.harvard.edu
Neurosurgical Service MGH, Harvard Medical School	http://neurosurgery.mgh.harvard.edu
JHMI-InfoNet, Johns Hopkins Medical Institutions	http://infonet.welch.jhu.edu
OncoLink, University of Pennsylvania	http://cancer.med.upenn.edu
CliniWeb, Oregon Health Sciences University	http://www.ohsu.edu/cliniweb/
HospitalWeb, Department of Neurology at MGH	http://neuro-www.mgh.harvard.edu/hospitalweb.shtml
**1.2 Government Original:**	
Health Services Technology Assessment Texts	http://text.nlm.nih.gov
National Center for Biotechnology Information	http://www.ncbi.nlm.nih.gov, http://www3.ncbi.nlm.nih.gov/omim/
National Institutes of Health Funding Opportunities	http://www.nih.gov/grants/
CDC Morbidity and Mortality Weekly Report	http://www.cdc.gov/epo/mmwr/mmwr.html
CDC National Center for Health Statistics	http://www.cdc.gov/nchswww/default.htm
**1.3 Commercial Original:**	
Medscape	http://www.medscape.com
PharmInfoNet	http://pharminfo.com
Galaxy directory	http://galaxy.einet.net
Galaxy directory	http://www.einet.net
Argus Clearinghouse (ACH)	http://www.clearinghouse.net
**1.4 Organizational original:**	
Medical Matrix	http://www.medmatrix.org/index.asp/
ExPASy molecular biology WWW server	http://expasy.hcuge.ch
Genome Database (GDB)	http://gdbwww.gdb.org
Cyber Science, NIH Molecular Biology Home Page	http://molbio.info.nih.gov
Chemical Abstracts Service (CAS)	http://info.cas.org
Foundation Center	http://fdncenter.org
CancerNet, National Cancer Institute (NCI)	http://cancernet.nci.nih.gov
**2. Universities and Institutes:**	
**2.1 Universities:**	
University of Pittsburgh	http://www.pitt.edu
Oregon Health Sciences University	http://www.ohsu.edu
Emory University	http://www.cc.emory.edu
University of Pennsylvania	http://www.upenn.edu
Health Information Research Unit at McMaster University	http://hiru.mcmaster.ca
**2.2 Hospitals and Medical centers:**	
Center for Molecular Medicine, Emory University School of Medicine	http://www.gen.emory.edu
Virtual Hospital ®, University of Iowa	http://indy.radiology.uiowa.edu
Virtual Hospital ®	http://www.vh.org
University of Pennsylvania Health System	http://www.med.upenn.edu
Massachusetts General Hospital	http://www.mgh.harvard.edu
**2.3 Libraries:**	
University of Iowa Libraries	http://www.lib.uiowa.edu
ROGER Catalog of UCSD Libraries	http://roger.ucsd.edu
The University of Michigan University Library	http://www.lib.umich.edu
National Library of Medicine	http://www.nlm.nih.gov
Agency for Toxic Substances and Disease Registry	http://atsdr1.atsdr.cdc.gov:8080
National Institutes of Health (NIH)	Gopher://gopher.nih.gov
Library of Congress	http://lcweb.loc.gov
**3. Organizations and Societies**	
Centers for Disease Control and Prevention	http://www.cdc.gov
American Medical Association	http://www.ama-assn.org
National Institutes of Health (NIH)	http://www.nih.gov
Association of American Medical Colleges	http://www.aamc.org
World Health Organization	http://www.who.ch, http://www.who.int
Federation of American Societies for Experimental Biology	http://www.faseb.org
The National Science Foundation	http://www.nsf.gov
U. S. Census Bureau	http://www.census.gov
U.S. Environmental Protection Agency	http://www.epa.gov
The Department of Health and Human Services	http://www.os.dhhs.gov
Oak Ridge National Laboratory	http://www.ornl.gov
Center for Food Safety & Applied Nutrition	http://vm.cfsan.fda.gov
Food and Drug Administration	http://www.fda.gov
Health Care Financing Administration	http://www.hcfa.gov
U.S. Government Printing Office	http://www.access.gpo.gov
American Psychological Association	http://www.apa.org
International Food Information Council Foundation	http://ificinfo.health.org
European Bioinformatics Institute (EBI)	http://www.ebi.ac.uk
U.S. Department of Education	http://www.ed.gov
Occupational Safety and Health Administration	http://www.osha.gov
National Academy of Sciences	http://www2.nas.edu
National Research Council	?????????????????
National Academy of Engineering	?????????????????
Institute of Medicine	?????????????????
**4. Search Engines:**	
Yahoo search engine	http://www.yahoo.com
Lycos search engine	http://www.lycos.com
Webcrawler search engine	http://webcrawler.com
Altavista search engine	http://www.altavista.digital.com
Excite search engine	http://www.excite.com
Hotbot search engine	http://www.hotbot.com
Infoseek search engine	http://www2.infoseek.com
Magellan search engine	http://www.mckinley.com
**5. Commercial Companies:**	
SLACK Incorporated	http://www.slackinc.com
P\S\L Consulting Group Inc	http://www.pslgroup.com
Community of Science, Inc	http://www.cos.com
Merck & Co., Inc	http://www.merck.com
Deja News, Inc	http://www.dejanews.com
RAMS-FIE	http://web.fie.com
**6. Journals:**	
Journals of American Society for Microbiology	http://asmusa.edoc.com

Combining the results of the "first level of domains" analyses and the whole URLs analyses, we replaced some "first level of domain" with the whole URL expansion if it existed. From this analysis a guide to the most cited health sciences related web sites was determined. We hope this list might serve as a more complete listing of the core web sites on health care.

To further represent these health-related core web sites clearly, we classified these core web sites respectively by their main utility, original sites into 6 clusters (Table 5).

## Conclusions

Among the URLs cited by the selected academic medical institutions, almost 90% of the Top Level Domains (TLDs) are from the United States. Less than 10% come from the United Kingdom, Switzerland, Canada, Germany, Australia and the Netherlands. The number of remaining TLDs is less than 2%.

The first level domains are distributed according to Bradford's Law. There is a nucleus that contains the 78 most highly cited health sciences related web sites. These core web sites represented a broad field of information needs.

## Discussion

The identification of a core section of health-related web sites with bibliometrics method gives librarians and information scientists another approach to evaluate the web sites. While "core lists" of printed publications have their drawbacks, they are useful guides to help librarians and users to select publications. Similarly, lists of commonly linked-to WWW pages can provide suggestions as to important health-related sites and assist home-page compilers in selecting suitable and reliable links. It would be desirable to examine the home pages of all U.S. medical school libraries and to compare these results to those from the pages produced by medical school libraries in other English-speaking countries such as Canada, the United Kingdom and Australia.
